# Commentary on German *et al*.: Qualitative inquiry reduces lags in scientific discovery‐to‐practice by recognizing the living expertise of people who use drugs

**DOI:** 10.1111/add.70471

**Published:** 2026-05-11

**Authors:** Megan K. Reed

**Affiliations:** ^1^ Jefferson Center for Connected Care Thomas Jefferson University Philadelphia PA USA; ^2^ Department of Emergency Medicine, Sidney Kimmel Medical College Thomas Jefferson University Philadelphia PA USA; ^3^ College of Population Health Thomas Jefferson University Philadelphia PA USA

**Keywords:** addiction research, adulterants, emerging topics, ethnography, people who use drugs, qualitative methods

*Drug policy changes, an evolving supply, and shifting drug use practices can unfold rapidly and prove difficult to be documented by researchers in real time. Substantial harm can be avoided through rapid ‘on the ground’ qualitative research with people directly impacted to identify targeted interventions*.

In an unregulated drug supply, as clandestine labs quickly and efficiently manufacture new substances and analogs, adulterants and novel psychoactive substances like xylazine, nitazenes and others will continue to proliferate [1, 2, 3, 4]. When novel phenomena arise, the typical lag of the scientific inquiry to practice timeline slows our understanding and effective responses [5]. The study conducted by German *et al*. [6] advances our limited knowledge about xylazine‐associated wounds using qualitative methods in collaboration with people using fentanyl in Baltimore, MD, USA. Approaches such as theirs are the most effective way to understand the contextual aspects of xylazine adulteration at a time when scant literature can inform public health and clinical practice to better understand wound etiology and impacts. Qualitative inquiry in partnership with populations of people who use drugs informs the scientific community, complements existing research, and advances clinical and intervention implementation.

Perspectives from people using xylazine can better direct us towards what to study and investigate. Seemingly unrelated topics that emerge during standard qualitative interviews can aggregate into new themes warranting further focus and elaboration. Early identification provided by research participants to investigators can result in novel studies that may otherwise be delayed by more traditional quantitative approaches that do not yet inquire or capture data about what is currently unknown to the research community.

In this study, the participants described their lived theories of wound causality and everyday strategies to avoid xylazine‐associated wounds in community settings that are typically obscured from researchers, policymakers and clinicians. Participants, the people directly impacted, shared their lived experiences and allowed investigators to better understand the measured outcomes. Each party brings their own expertise; combining these viewpoints gives us a more holistic picture.

Perspectives described from an insider viewpoint are essential to inform practices by outsider actors who do not have lived experience [7]. Participants in this study provide recommendations to avoid xylazine‐associated wounds within their own communities. These may prevent the need for future clinical care or provide opportunities outside of clinical settings to supplement current clinical best practices for treating wounds, such as optimizing drug preparation sanitation, vein care and addressing co‐occurring conditions. Regarding diabetes mellitus, clinicians may otherwise not be aware that people with diabetes and xylazine‐associated wounds may expect slower healing times.

Informing, complementing and advancing knowledge are core missions of research. Qualitative approaches to achieve this mission should be applied systematically to emerging health issues, not only with the drug supply, but for any phenomena people who use drugs experience in their daily lives.

## AUTHOR CONTRIBUTIONS

**Megan K. Reed:** Conceptualization (lead); writing—original draft (lead).

## DECLARATION OF INTERESTS

The author has no financial or other relevant links to companies with an interest in the topic of this article.
